# Mr. Stephens' Views on the Subject of Hernia; Cases and Remarks

**Published:** 1829-10-01

**Authors:** 


### XLVIII.

### Mr. Stephens' Views on the Subject of Hernia.

In the last number of this Journal, page 109 *et seq.* we noticed an interesting work on Hernia by Mr. Stephens, and gave, or thought we gave, an even-handed measure of justice to the author. The following opinion appeared in our critique.

"No doubt can exist, nor indeed has at any time existed, that bowels confined by adhesions in hernial sacs will not go on with their natural functions so freely as when loose and floating in their native cavity. But the question is, will adhesions, *per se,* occasion a fatal obstruction to the office of the gut? Mr. Stephens says they will; and the issue is with him and his surgical brethren. For our own parts, we doubt whether such be the case, nor can we imagine that a patient will die from this cause alone. The case is very different when the adhesions are so arranged as to act like a stricture on the gut, or when the latter is so placed as, by being convoluted on itself, or in any other manner, to prevent the egress of matters from it cavity."

On the 24th of July, we received a letter from Mr. Stephens, in reference to the sentiments we have expressed in the preceding passage. Mr. Stephens observed, that he was "sure we would agree that it is the duty of a public journalist, if he should at any time express an opinion which he subsequently finds to be erroneous, or if he should dissent from the opinion of an author, whose judgment he should afterwards find right, to rectify his error as soon as possible, and admit readily any evidence tending to establish truth, whether it may be for or against his opinion." Certainly such is not only the *duty* of a journalist, but should always be his *inclination;* we can say most sincerely that it is our's. But we have no intention of indulging in what may pass for vanity and egotism; on the contrary, we shall hurry, without further preface, to the consideration of two cases brought forward in his letter by Mr. S. as corroborative, or rather confirmatory, of the soundness and healthiness of his views. Both are recorded in our valued contemporary, the London Medical Gazette.

*Case* 1. This occurred in the private practice of Mr. Vincent, and is related in the number of the Gazette, for the 18th of April, by Mr. Burnett, house-surgeon to St. Bartholomew's Hospital. We cannot abridge the account in any material degree, and perhaps it would be more satisfactory to all parties, to give it in the words of the narrator.

" A robust and healthy-looking woman, fifty-six years of age, who had been in the habit of making such exertions as her trade, which was that of a grocer, required—had never been the subject of hernia, nor afflicted with any other complaint than a prolapse of the uterus, which she had had for many years, and which never gave her more trouble than the mere inconvenience which it produced. She had been a widow for many years. Although she had been accustomed to lift weights, still, from the nature of her complaint, these could not necessarily be very great; nor was she at all conscious of having made use of any force which could have accounted for the formation of a hernia.

" On the 23d of March she directed Mr. Vincent's attention to an inflamed swelling, somewhat larger than a pigeon's egg, which took its seat rather upon than below Poupart's ligament of the left side, and a little to the inner side of the external ring. It was moveable, and presented very much the character of an inflamed gland. But this superficial swelling, for so I may call it, seemed to lie immediately upon another, which was deeper seated, and consequently more obscurely felt; though, when pressed upon, it gave considerable resistence to the finger, was perfectly immoveable, and excessively painful on being touched. She had observed this swelling only the day before, since which she had not had any motion from the bowels, but had been in a constant state of sickness, accompanied by hiccup. The abdomen all over was tender on pressure, but at the lower part of the left side she complained of its giving her acute pain. The pulse was quick and strong. Mr. Vincent took away about fourteen ounces of blood from the arm, which afterwards became very much cupped and buffed. Mr. V. then ordered that she should take repeated doses of Epsom salts; but the stomach rejected every thing; in consequence of which, enemata were repeatedly administered, but without moving the bowels. She continued restless through the night, and on the following day (24th), at two o'clock, there did not appear to be any alteration in the symptoms. She had retained no food. On directing her to cough, no impetus was communicated to the part, while she at the same time voluntarily observed, that it gave her no pain.

" Considering all the circumstances of the case—the excessive tenderness, the constant sickness, the hiccup, and the constipated state of the bowels—Mr. Vincent thought (and those gentlemen who were present agreed in his opinion) that it would be advisable not to defer the operation of cutting down upon the tumor. An incision was accordingly made, an inch and a half or two inches long, commencing just below the external ring, and passing downwards and a little inwards upon the tumor. After the fat was divided, the first thing observed was an inflamed and enlarged gland, which seemed to block up the wound. This was divided, when another tumor came into view, corresponding to the one which had been felt beneath. The operator raised the gland, and cutting horizontally upon this last tumor, which was the size of the tip of the finger, expected by so doing to enter the sac of the hernia. The part had a black appearance, and looked like a sac in which the circulation had been obstructed by stricture above. The division of this, however, only exposed another covering beneath, to which it was very firmly adherent. Mr. V. now raised a portion of this last covering with the forceps, as he had done the former, and, making a similar horizontal cut, when about an ounce of a turbid yellow urinous-smelling fluid gushed out. He now thought he had opened the sac of the hernia, and, after having enlarged the aperture, introduced the point of the index finger, and felt for the strictured gut, but there was none to be found—the sac was quite empty. He desired the patient to cough, but no impetus was given by so doing to the tumor, and the sac appeared to have no connexion with the abdomen; but the opening under Poupart's ligament was plainly to be felt, and by passing a director upwards, in the direction of the sac, it was observed to enter the cavity of the abdomen.

"The patient did not express herself relieved by the operation, though the sickness left her for a time, and as there was no intestine or omentum to reduce, the wound was immediately closed by a ligature and some pieces of strap, after which she was placed in bed. Fourteen ounces of blood were drawn from the arm, the pulse being sharp; she then felt faint, but slept for about four hours. At the expiration of this time an injection was given, but it produced no effect, and she remained restless, though free from sickness.

"25th.—The sickness had not returned, and she was now directed to take calomel and colocynth by the mouth; but after these had been repeated a few times, the sickness returned, without their producing any evacuation by the bowels. The blood which had been drawn yesterday was not cupped or buffed; but there was still the pain and tenderness of the abdomen, and the pulse was beating about 90. Several injections were now repeated, but to no purpose; and in the evening she brought up, by vomiting, a great quantity of matter which had the appearance, and she said tasted, of the injection.

"26th.—The night was disturbed by sickness, and she continued to bring up matter of the same character. Altogether she had vomited about three quarts of fæcal matter. The abdomen was more tender. Two large blisters were now placed on each side of the abdomen; she felt considerably relieved by them, but this relief was not permanent—nor, perhaps, depending entirely upon the blisters. Another injection was administered, which was the only one which seemed to be at all efficacious; it produced an evacuation of a quantity of hardened scybala, and the relief was great for the time.

"27th.—The sickness had gone off, and she slept occasionally through the day. The pulse was the same in frequency, but rather smaller. As there had been no evacuation from the bowels, she was ordered to take three grains of calomel and twelve of jalap.

"28th.—No evacuation from the bowels. The stomach could retain small quantities of farinaceous food, but she seemed lower, and her pulse was materially weaker. In the evening the sickness returned with greater violence; her anxiety was very great. An injection was given, which was returned without any thing. She was ordered some pills containing calomel and opium, but the night was passed in increased restlessness and anxiety, and she died early the next morning.

"The examination was conducted by Mr. Vincent, in the presence of one of the female relatives. On opening the cavity of the abdomen, neither the peritoneum nor intestines were, to appearance, at all inflamed. Towards the lower portion of the ileum, in tracing it down, there was seen to be about an inch of its long diameter adherent to the neck of the sac, but only by about half an inch of the calibre of the intestine; which part was so firmly attached as to look as if it had been nipped. The inner coat about this part of the intestine was ulcerated, and there was a slight inflammation around; but the channel of the bowel was perfectly free, and its circumference opposite to the part which was nipped not at all inflamed."

When we say that we are not convinced by the foregoing case, Mr. Stephens will probably consider us as unbelieving as the brethren of Dives in the parable. We do not see, however, why the mere adhesion for the space of an inch should be singled out as the cause of death, whilst the *nipping* of the gut and the ulceration of the inner coat are passed, or to be passed, in silence. Surely these morbid appearances could not have been produced by the adhesion, and if so, where shall we look for *their* cause? It appears to us that most probably the inflamed and altered portion of the gut, not comprising the whole of its circumference, was at one time involved in the sac and constricted by its neck. This is the most feasible solution of the difficulty, but even allowing it to be a true one we admit that the case is still obscure. At all events, we do not think it fair to advance the adhesions as the whole and sole cause of the symptoms and issue.

*Case* 2. This is related by Mr. Hewson in the Med. Gazette for July 18th, in a communication to the editor. Like the preceding case, it shall be given in the unadulterated words of the communicant, in order that our readers may perceive our wish not to blink the question.

"John Westfield, aged 55, of spare habit of body, admitted late in the evening of Thursday, May 28th, 1829, under the care of my colleague Mr. Boot, senior surgeon to the Lincoln County Hospital, for a scrotal hernia of the left side. The tumor is of a pyramidal shape; the integument covering the herniary tumor has a natural appearance; is inelastic; but the taxis produces a gurgling noise, as in intestinal hernia; considerable pressure upon the tumor occasions no pain; he has hiccup, and eructations of air, with cold extremities. He states that he has been affected with a rupture for the last thirty years, which has usually been of the size of a pullet's egg. Early on Monday morning, the 18th, whilst in the act of throwing stones into a cart, he perceived the hernia suddenly to enlarge, more so than he had ever known it before: this was followed by slight pain of abdomen, and nausea: he has passed no stool since the Sunday (17th) preceding the descent of the hernia; prior to admission into the hospital, he vomited daily, and had pain of abdomen, with fever.

"He was immediately bled to 16 oz. which produced fainting; but the taxis failed to make any impression on the tumor. Cathartic clysters were given, with castor oil, which produced two slight evacuations, and cold was directed to be applied to the swelling.

"May 30th.—Restless night. Pulse 80, small and thready. He continues to vomit, and the egesta have a decidedly fæcal character; no further evacuation by the anus; very little tension of the abdomen, on which pressure produces little or no uneasiness, but on the neck of the sac it produces pain; cold had been applied to the tumor, but was omitted at the patient's request, in consequence of its causing considerable pain; body and extremities cold. Ordered to have a cordial mixture, and cathartic clysters every four hours.

"31st. Bad night. Pulse 80, very small and thready. Hands and face very cold. Generally lies upon his left side; when lying on his back, he invariably begins to vomit; the clysters return unaltered.

"June 1.—Has passed a very restless night. Pulse 80, very feeble. Constant eructations of air. Complains of more pain across the region of the bladder, and along the course of the transverse arch of the colon. Vomits much stercoraceous matter. No stools.

"2d.—Has had a better night, with less vomiting. Can take nothing but a little wine and water.

"3d.—In every respect the same. Has had a purging stool. Pulse 80, small and thready.

"4th.—Slept but little: was not restless. Pulse 84, scarcely perceptible. He constantly lies upon his left side, with the trunk bent. He is gradually sinking.

"5th.—Died at 7 o'clock this morning.

"*Post Mortem Examination*, 5 *hours after death*.—On opening the cavity of the abdomen, sero-purulent fluid was observed between the agglutinated folds of inflamed intestine; the great omentum twisted together, as if slightly encircled with a string; the whole of the intestinal canal exhibiting marks of inflammation, and distended with air and fæces. The arch, ascending and descending portions of the colon, were more particularly inflamed and distended; the peritoneum lining the abdominal muscles not inflamed; the common integuments of the herniary tumor quite natural in appearance; the fascia superficialis and sac much thickened, the latter contained very little fluid; the sac contained a considerable portion of omentum, about the size of a turkey's egg: this was healthy in appearance, slightly inflamed, and adherent to the mouth of the sac only. No intestine was found in the sac, but a portion of the ileum had become adherent to the omentum by its peritoneal surface at the mouth of the sac. This portion of intestine had a very dark appearance, but it regained its natural colour after immersion in spirits of wine. The calibre of the gut was not at all diminished or interrupted. The hernia was congenital, the omentum in the sac being in contact with the testicle.

"As the omentum had a healthy appearance, I am of opinion that the adhesions were formed in consequence of the last descent of omentum, and that this case distinctly points out the danger of allowing omentum to remain for any length of time in the sac of a hernia.

"In the above case there was no stricture or diminution of the calibre of the gut, but a simple adhesion between the omentum and intestine, yet this proved amply sufficient to produce derangement of the functions of the alimentary canal, and ultimately death, with all the symptoms of incarcerated hernia."

Now really we think this case more opposed to Mr. Stephens' views than the former. A man of 55, affected for thirty years with a rupture some of it apparently irreducible, receives a strain, perceives the hernia suddenly enlarge, and is instantly attacked by symptoms of strangulation or peritoneal inflammation. Little seems to be done for ten days, when he enters the hospital with irreducible tumour, obstinate constipation, vomiting, abdominal tenderness, small thready pulse, and hiccup. No relief can be afforded either locally or generally, and eight days after admission he dies. On examination the evidences of general peritoneal inflammation are apparent, and omentum is contained in the sac and adherent to its neck, where also it had contracted an adhesion to a portion of the ileum.

We cannot see a shadow of justice for assuming that the adhesions were the cause of the fatal issue of the case, nor indeed that they had any thing to do with it. Most probably the connexion of the omentum to the neck of the sac had existed as long as the rupture itself, and yet not a particle of danger did it give rise to, during the whole of that period. It is assuming too much to assert that the omento-intestinal adhesion produced the general inflammation; on the contrary, we believe that the general inflammation was the cause of the adhesion. We are told in the commencement of the account of the examination, that folds of intestine were *agglutinated* together. There is nothing unusual in this, but why should *their* adhesions be overlooked? It would be droll pathology to conclude, on examining a case of peritonitis, that the adhesions of the intestinal convolutions which we commonly find, were the *cause* of the inflammation. And yet it is precisely this kind of argument, *à posteriori*, that would lay the peritonitis, in the present instance, to the door of the adhesion between the omentum and the ileum.

Thus, then, we have fully noticed the cases to which Mr. Stephens has directed our attention. The opinions we have drawn from them differ from his, but still Mr. Stephens may be in the right. Not filling the chair of St. Peter we cannot lay claim to infallibility, but we hope that none will blame us for candidly avowing the sentiments we entertain on this interesting subject. Our brethren will decide on the justice or fallacy of Mr. Stephens' views, and whichever shall ultimately be found to be the case, will be equally satisfactory to us, for truth must be the gainer.

